# Supplementary material for: Extracting patient-level data from the electronic health record: Expanding opportunities for health system research
Source: PLoS One. 2023 Mar 10;18(3):e0280342. doi: 10.1371/journal.pone.0280342 (PMC10004557; doi:10.1371/journal.pone.0280342)
Supplement: S1 Table — (DOCX) [file pone.0280342.s001.docx]

**Supporting Information**

# S1 Table. ILD Diagnostic Codes and Procedure Codes

| **ICD-9-CM Code** | **ICD-10 Code** | **Description** |
| --- | --- | --- |
| 135, 517.8 | D86.0, D86.2, D86.9 | Sarcoidosis, Sarcoidosis of lung with sarcoidosis of lymph nodes |
| 515 | J84.89, J84.9,  J84.10,  J84.112,  J84.17, | Interstitial Pulmonary Fibrosis, Post-inflammatory pulmonary fibrosis, nonspecific interstitial pneumonia, acute interstitial pneumonia, chronic interstitial pneumonia, interstitial lung disease |
| 516.30 | J84.111 | Idiopathic interstitial pneumonia, not otherwise specified |
| 516.31 | J84.112 | Idiopathic pulmonary fibrosis, Familial idiopathic pulmonary fibrosis |
| 516.32-516.37 | J84.113-  J84.117, J84.2 | Idiopathic nonspecific interstitial pneumonia, acute interstitial pneumonia, respiratory bronchiolitis interstitial pneumonia, idiopathic lymphoid interstitial pneumonia, cryptogenic organizing pneumonia, and desquamative interstitial pneumonia |
| 495.0, 495.2,  495.5, 495.8,  495.9 | J 67.0, J67.2,  J67.5, J67.8, J67.9 | Extrinsic allergic alveolitis, Hypersensitivity Pneumonitis |
| 500 | J60 | Coal workers’ pneumoconiosis |
| 501 | J61 | Asbestosis |
| 502 | J62.8 | Pneumoconiosis due to other silica or silicates |
| 503 | J63.4, J63.6 | Pneumoconiosis due to other inorganic dust |
| 504 | J66.8 | Pneumonopathy due to inhalation of other dust |
| 505 | J64 | Pneumoconiosis, unspecified |
| 516.8 | J84.09 | Other specified alveolar and parietoalveolar pneumonopathies |
| 516.9 | J84.09 | Unspecified alveolar and parietoalveolar pneumonopathies |
| 517.2, 710.1 | M34.81 | Lung involvement in systemic sclerosis |
| 714.81 | M05.10 | Rheumatoid lung |
| 518.89 | J98.4 | Chronic restrictive lung disease, drug-induced lung disease, occupational lung disease, lung disease due to connective tissue disorder |
| 518.89, 710.9 | J98.4, M36.8 | Lung disease due to connective tissue disorder |
| 515, E980.5 | J70.3, J70.4 | Chronic drug-induced interstitial lung disorders,  Drug-induced interstitial lung disorders, unspecified |
