# Supplementary material for: Extracting patient-level data from the electronic health record: Expanding opportunities for health system research
Source: PLoS One. 2023 Mar 10;18(3):e0280342. doi: 10.1371/journal.pone.0280342 (PMC10004557; doi:10.1371/journal.pone.0280342)
Supplement: S2 Table — Percentages of the ILD Cohort receiving care in each of the KPNC regions. Values are No. (%). (DOCX) [file pone.0280342.s002.docx]

**Supporting Information**

# S2 Table. Primary KPNC Facility

| Geographic Location | Total N = 5399 |
| --- | --- |
| Diablo/Napa | 1003 (19%) |
| East Bay/GSAA | 719 (13%) |
| Fresno/Central Valley | 429 (8%) |
| Sacramento Valley | 955 (18%) |
| Santa Clara/San Jose | 652 (12%) |
| West Bay | 838 (16%) |
| Missing | 803 (15%) |

Percentages of the ILD Cohort receiving care in each of the KPNC regions. Values are No. (%).
