# Supplementary figures and images for: Extracting patient-level data from the electronic health record: Expanding opportunities for health system research
Source: PLoS One. 2023 Mar 10;18(3):e0280342. doi: 10.1371/journal.pone.0280342 (PMC10004557; doi:10.1371/journal.pone.0280342)

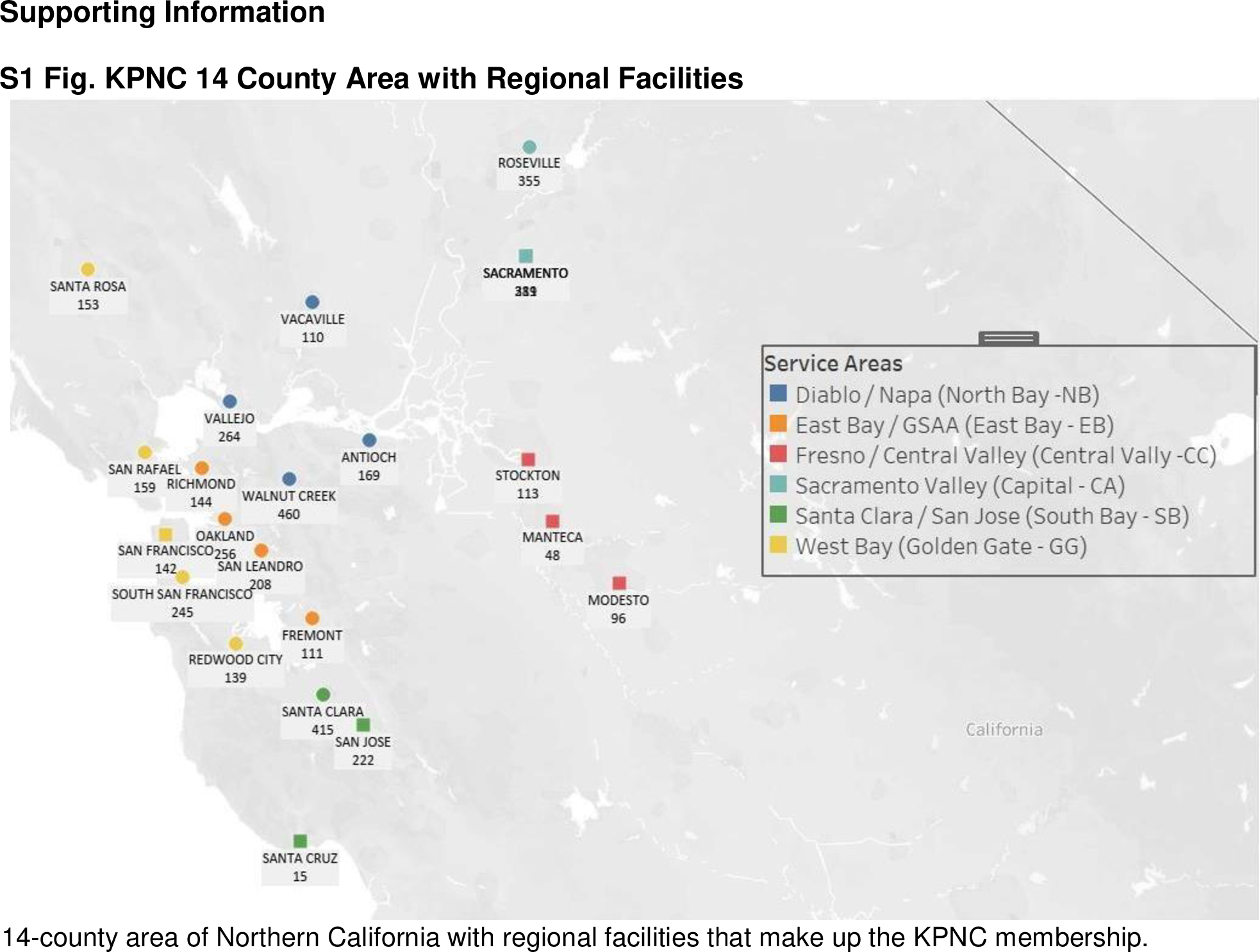

Supplement: S1 Fig — 14-county area of Northern California with regional facilities that make up the KPNC membership. (TIF) [file pone.0280342.s003.tif]

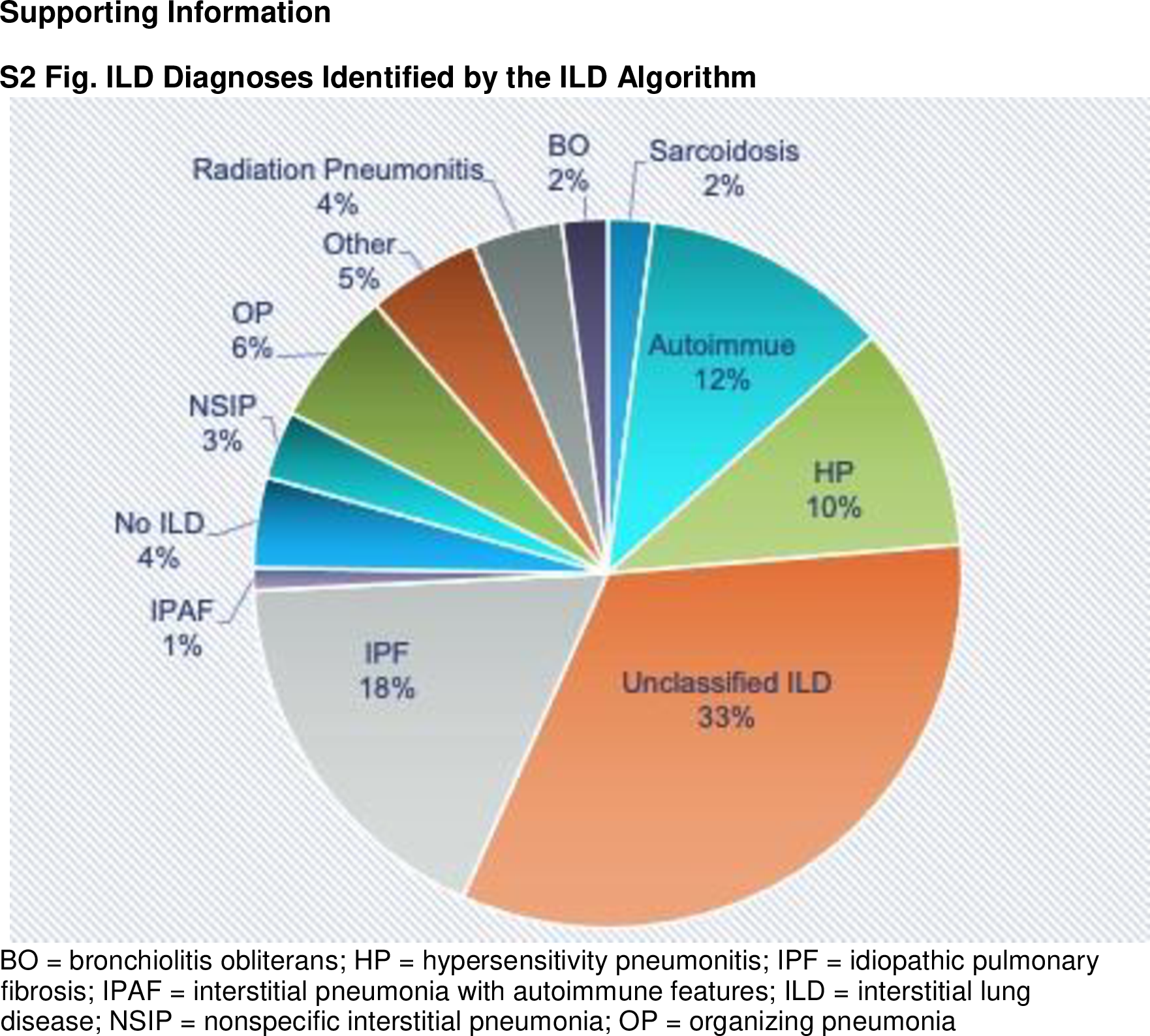

Supplement: S2 Fig — BO = bronchiolitis obliterans; HP = hypersensitivity pneumonitis; IPF = idiopathic pulmonary fibrosis; IPAF = interstitial pneumonia with autoimmune features; ILD = interstitial lung disease; NSIP = nonspecific interstitial pneumonia; OP = organizing pneumonia. (TIF) [file pone.0280342.s004.tif]

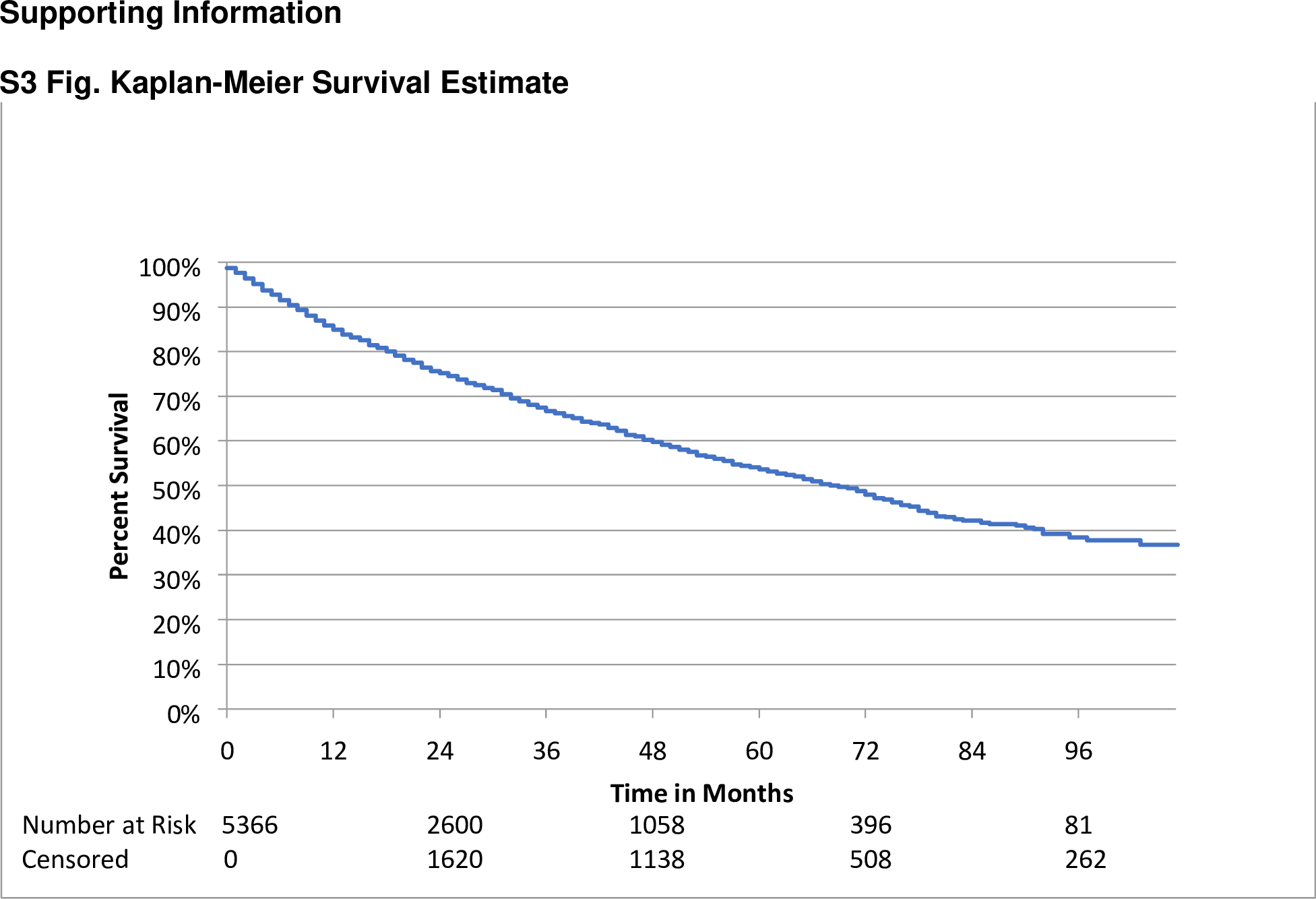

Supplement: S3 Fig — Survival estimate of the full ILD cohort. The number of patients at risk over time decreased due to a combination of lost-to-follow up, right censoring and death. (TIF) [file pone.0280342.s005.tif]
