# Supplementary material for: Extracting patient-level data from the electronic health record: Expanding opportunities for health system research
Source: PLoS One. 2023 Mar 10;18(3):e0280342. doi: 10.1371/journal.pone.0280342 (PMC10004557; doi:10.1371/journal.pone.0280342)
Supplement: Attachment — Submitted filename: Response to Reviewers_9.19.docx [file pone.0280342.s006.docx]

Dear Paula Katrina A. Maderazo and Reviewers,

We want to express our appreciation to the reviewers and editors for considering our manuscript entitled “Extracting Patient-Level Data from the Electronic Health Record: Expanding Opportunities for Health System Research”. We thank the editors for their comments drawing attention to journal requirements. We made targeted revisions to directly address these issues.

Please see the comments from the editors with our responses below.

Sincerely,

Erica D. Farrand, M.D.

University of California, San Francisco

Division of Pulmonary, Critical Care

Department of Medicine, UCSF School of Medicine

***JOURNAL REQUIREMENT 1:*** Please provide additional details regarding participant consent. In the ethics statement in the Methods and online submission information, please ensure that you have specified (1) whether consent was informed and (2) what type you obtained (for instance, written or verbal, and if verbal, how it was documented and witnessed). If your study included minors, state whether you obtained consent from parents or guardians. If the need for consent was waived by the ethics committee, please include this information.

If you are reporting a retrospective study of medical records or archived samples, please ensure that you have discussed whether all data were fully anonymized before you accessed them and/or whether the IRB or ethics committee waived the requirement for informed consent. If patients provided informed written consent to have data from their medical records used in research, please include this information.

Once you have amended this/these statement(s) in the Methods section of the manuscript, please add the same text to the “Ethics Statement” field of the submission form (via “Edit Submission”).

***RESPONSE TO REQUIREMENT 1:*** Our full ethics statement was previously included in the online submission form but not in the methods section. The statement has been added to the methods section and now reads:

*Institutional review boards at the University of California San Francisco (#14-15459), and the KPNC Division of Research (#CN-15-2126-H_05) approved the study protocol. The primary dataset was deidentified prior to access. A subset of patient records was identified for algorithm validation. The IRB waived the requirement for informed consent for this retrospective study of medical records.*

***JOURNAL REQUIREMENT 2:*** Thank you for stating the following in your Competing Interests section: “Drs. Erica Farrand, Michael Guarnieri, George Minowada, Mr. Lawrence Block, and Ms. Mei Lee have declared that no competing interests exist. Dr. Harold Collard has read the journal's policy and has the following competing interests: personal fees from Advanced Medical, Polarean and WebMD, and grants from Pulmonary Fibrosis Foundation outside of the submitted work.”

Please complete your Competing Interests on the online submission form to state any Competing Interests. If you have no competing interests, please state "The authors have declared that no competing interests exist.", as detailed online in our guide for authors at [http://journals.plos.org/plosone/s/submit-now](https://urldefense.com/v3/__http:/journals.plos.org/plosone/s/submit-now__;!!LQC6Cpwp!pBhkGOYv8yFoJ-XN53piI7-H996lZRmC5ON3nwAkCWUJiERXpKovESC8KFvJNhl5Vtm1WbCLmFHp6HB0txsh$)

 This information should be included in your cover letter; we will change the online submission form on your behalf.

***RESPONSE TO REQUIREMENT 2:*** The cover letter has been updated to include the Competing Interests statement in accordance with the online author guidance It now reads:

*We have read the journal’s policy and one of the authors of this manuscript (Dr. Harold Collard) has the following competing interests: personal fees from Advanced Medical, Polerean, and WebMD, and grants from Pulmonary Fibrosis Foundation outside of the submitted work. These entities provided no funding and had no role in the design, preparation, or submission of this manuscript. The competing interest does not alter our adherence to PLOS ONE policies on sharing data and materials.*

We were unable to update the online submission form to reflect this change but appreciate you modifying it our behalf.
